# Recurrent Sclerema in a Young Infant Presenting with Severe Sepsis and Severe Pneumonia: An Uncommon but Extremely Life-threatening Condition

**DOI:** 10.3329/jhpn.v31i4.20053

**Published:** 2013-12

**Authors:** Farzana Afroze, Mark A.C. Pietroni, Mohammod Jobayer Chisti

**Affiliations:** ^1^Clinical Services Centre, icddr,b, GPO Box 128, Dhaka 1000, Bangladesh; ^2^Centre for Nutrition & Food Security (CNFS), icddr,b, GPO Box 128, Dhaka 1000, Bangladesh

**Keywords:** Blood transfusion, Malnutrition, Sclerema, Severe sepsis, Bangladesh

## Abstract

A one month and twenty-five days old baby girl with problems of acute watery diarrhoea, severe dehydration, severe malnutrition, and reduced activity was admitted to the gastrointestinal unit of Dhaka Hospital of icddr,b. The differentials included dehydration, dyselectrolytaemia and severe sepsis. She was treated following the protocolized management guidelines of the hospital. However, within the next 24 hours, the patient deteriorated with additional problems of severe sepsis, severe pneumonia, hypoxaemia, ileus, and sclerema. She was transferred to the Intensive Care Unit (ICU). In the ICU, she was managed with oxygen supplementation, intravenous antibiotics, intravenous fluid, including a number of blood transfusions, vitamins, minerals, and diet. One month prior to this admission, she had been admitted to the ICU also with sclerema, septic shock, and urinary tract infection due to *Escherichia coli* and was discharged after full recovery. On both the occasions, she required repeated blood transfusions and aggressive antibiotic therapy in addition to appropriate fluid therapy and oxygen supplementation. She fully recovered from severe sepsis, severe malnutrition, ileus, sclerema, and pneumonia, both clinically and radiologically and was discharged two weeks after admission. Consecutive episodes of sclerema, resulting in two successive hospitalizations in a severely-malnourished young septic infant, have never been reported. However, this was managed successfully with blood transfusion, broad-spectrum antibiotics, and correction of electrolyte imbalance.

## INTRODUCTION

Sclerema is defined as diffuse hardening of subcutaneous tissue, which usually spreads very rapidly to underlying structures; the skin in the involved areas cannot be picked up, and the subcutaneous tissue seems bound down to subjacent muscle and bone (1). It is a life-threatening condition which occurs in young infants with overwhelming sepsis, hypothermia, hypoxaemia, hyponatraemia, acidosis, and hypocalcaemia (1). The reported case fatality from sclerema in different series ranged from 30% to 100% (2-4), and deaths usually occur within hours to days of onset of the condition (5). Although sclerema no longer exists in developed countries, it still develops in young infants in resource-poor countries, like Bangladesh. However, the magnitude of the problem is still unknown. It is more frequently observed in neonates but has also been reported in older infants (1,2,4). Sclerema is characterized by the deposition of crystals, primarily of triglycerides, in the subcutaneous fat that can be determined by x-ray diffraction; however, some studies reported subcutaneous fibrosis, not crystallization of fat contents, as the conspicuous feature (3,6,7). As the progression of sclerema is very rapid, aggressive management is a *sine qua non* to reduce the morbidity and deaths from this condition (1,8). Important aspects of management of sclerema include intensive therapy for sepsis, circulatory support, blood transfusion, and correction of fluid and electrolyte imbalance (1,4,8). Although no randomized trials have been done to evaluate the efficacy of any management of sclerema till date, transfusion of fresh blood with or without exchange has been shown to have dramatic response in young infants with sclerema (9-11). We found no published data, in which there were two episodes of sclerema in two separate hospital admissions in the same child. Here, we present a surviving case of a one month and 25 days old infant who experienced two episodes of sclerema in two separate admissions and who also had septic shock and severe malnutrition.

## CASE REPORT

A one month and twenty-five days old baby girl from a low-income family, with monthly family income of TK 5,000, living in a slum area of Dhaka city was brought to Dhaka Hospital of icddr,b on 6 September 2011. She was the 4th issue of her non-consanguineous parents. Her mother was a 35 years old healthy woman, and her antenatal period was uneventful. The baby was delivered at home at full term but her birthweight and height were not recorded. She was fed diluted formula milk, along with breastmilk since birth. The girl was not vaccinated against any infectious diseases. She did not have a history of contact with any person known or suspected to have tuberculosis, and her family members were healthy. The parents were non-literate, the father was a watchman, and the mother was a housewife. The baby girl was admitted with a history of watery diarrhoea and occasional vomiting for 7 days, low-grade fever and breathing difficulty for 3 days, and reluctance to eat for 2 days. Her stool frequency was 5-6 times per day, copious in amount but there was no visible blood or mucous. There was no convulsion or rash. She received properly-prepared oral rehydration salts (ORS) solution at home.

One month ago, she had been admitted into the same hospital with similar complaints. At that time, she was diagnosed as a case of neonatal sepsis complicated by septic shock and sclerema. Her blood culture grew coagulase-negative *Staphylococcus* (CNS), and urine culture revealed *Escherichia coli* (colony count >10^5^). She received two boluses of intravenous fluid resuscitation (20 mL/kg/bolus), intravenous antibiotics (ampicillin 200 mg/kg/day and gentamicin 5 mg/kg/day), inotropic support, and two units of blood. Fourteen days later, she had fully recovered and was discharged with a follow-up plan.

On second admission, the girl was lethargic and mildly pale, and her rectal temperature was 37.8 ˚C. Her radial pulse was feeble, and blood pressure was unrecordable, capillary refilling time was 5 seconds, and extremities were cold. She had severe dehydration assessed according to the Dhaka Method (12). The infant weighed 1.8 kg, with a length of 45 cm, and her anthropometric measurements showed a z-score of <-3 for all three indices—weight-for-age, weight-for-length, and length-for-age of the WHO median. She did not have cyanosis, jaundice, oedema, or clubbing. The girl was tachypneic, with a respiratory rate of 60 per minute. She had lower chest wall in-drawing but no nasal flaring, head nodding, stridor, wheezing, or grunting respiration. Her breathing sounds were vesicular with no added sounds (rales, ronchi, or pleural rub). She had oral thrush with angular stomatitis, the abdomen was mildly distended with active bowel sound, and there was no organomegaly. Other systemic examination of the infant was unremarkable.

Her blood glucose, measured at the bedside, was 3.8 mmol/L, and her arterial oxygen saturation (SPO_2_) was 97% in room air (WHO defines SPO_2_ of <90% at sea-level as hypoxaemia).

The initial problems were: (a) acute watery diarrhoea with severe dehydration; (b) severe malnutrition; (c) reduced activity (the differentials included dehydration, dyselectrolytaemia, severe pneumonia, and severe sepsis).

The laboratory workup included: complete blood count, blood culture and sensitivity, serum electrolytes, creatinine, rectal swab culture for isolation and identification of enteric pathogens, urine culture and sensitivity, and a chest x-ray to confirm pneumonia. Initially, intravenous rehydration therapy was started for correction of severe dehydration by using half-strength acetate with 5% dextrose with 13 mmol/L of potassium chloride. Subsequently, she was managed as a case of severe sepsis; a fluid bolus was given (@ 20 mL/kg) following the standard sepsis guideline of the hospital; wide antimicrobial coverage was initiated using parenteral ampicillin and gentamicin. The infant was also provided with micronutrients, vitamins and minerals, and feeds according to the hospital's standard protocolized management of severe malnutrition and diarrhoea.

Her total WBC was 20,000/mm^3^, with 50% neutrophils, no bands, and 48% lymphocytes. Serum creatinine was 118.3 umol/L, TCO_2_ was 6.9 mmol/L, serum sodium was 143 mmol/L, and serum potassium level was 4.74 mmol/L, and total calcium was 1.8 mmol/L. The chest x-ray revealed patchy opacities in the right perihilar and pericardiac areas.

Twelve hours after admission, she was still lethargic, with little voluntary movement. There was a hardening of the skin and subcutaneous tissues of her both buttocks and thighs. She voided urine 8 hours after admission. Twenty-four hours later, the baby became extremely listless and hypotensive (BP 60/30 mmHg, respiration rate 60/min, pulse rate 150/min, low volume, capillary refilling time 4 sec, with cold extremities, SPO_2_ 88% in room air). She was transferred to the Intensive Care Unit of the hospital. She looked pale, puffy, and oedematous, even though pitting oedema was absent. Her hands and feet looked waxy white, and the limbs were tallow-hard. The sclerema became more extensive involving both buttocks and extremities, and, to a lesser extent, abdomen, chest, and face (ascending type). It did not involve the palms, soles or genitalia. Her reflexes were depressed, the abdomen became distended and tense, and she was not tolerating oral feeds. At that time, the problem list was revised to severe sepsis, very severe pneumonia, ileus, and sclerema. In the Intensive Care Unit, ampicillin and gentamicin were substituted for ceftazidime and amikacin as the patient's condition had deteriorated; another fluid bolus (20 mL/kg) was given, after which her blood pressure recovered, and she was given nothing orally.

In spite of adequate antimicrobials and supportive therapies, the baby's condition progressively deteriorated. At this point, forty-eight hours after admission, it was decided that she might benefit from blood transfusion. So, a fresh blood transfusion was arranged (10 mL/kg), and she received a transfusion daily in the next five days. On the fourth day of hospital stay after her second transfusion, her general condition started to improve; she became alert but the sclerema persisted. Feeding through nasogastric tube was started and well-tolerated. A marked improvement was noticed at the end of the fifth transfusion; the sclerema had softened, and the skin looked pink. The baby became more active and subsequently breastfeeding was re-established. All cultures (blood, urine, and stool) were negative. Parenteral antimicrobials were continued for 10 days. The child was discharged after full resolution of the sclerema two weeks after admission, and her parents were counselled on breastfeeding, immunization, and subsequent follow-up.

## DISCUSSION

The major observation of this case report is the development of sclerema in association with septic shock on two occasions in two successive hospitalizations in this severely-malnourished young infant who was also treated successfully with blood transfusions in addition to appropriate antibiotics, intravenous fluid, inotropes, correction of electrolyte imbalance, supplementation of micronutrients, vitamins and minerals, and therapeutic diet (13).

We do not have a ready explanation why this infant had two consecutive episodes of sclerema in two successive admissions in the hospital. It is likely that severe malnutrition and lack of breastfeeding both increased the risk of severe infection (14) in this infant. In both the admissions, the child had the co-morbidity of severe acute malnutrition and severe sepsis, which are strong risk factors for the development of sclerema (1,5).

Fat in young infants and neonates has higher saturated to unsaturated fatty acid ratio than in adults probably due to underdevelopment of the enzyme systems involved in desaturation of palmitic and stearic acid (3). Severe sepsis is associated with ineffective peripheral circulation, which might alter the saturated to unsaturated fatty acid ratio and contribute to hardening of subcutaneous fat and thickening of collagen fibres (1,5). Moreover, the infant had hypoxaemia and metabolic disturbances, which could further increase the ratio possibly by enzymatic alterations, resulting in precipitation of fatty acid crystals, primarily of triglycerides within the lipocytes, leading to the dramatic clinical findings of sclerema (5,15). In addition to severe sepsis and severe malnutrition, this girl had a urinary tract infection caused by *E. coli*, which might have contributed to the development of sclerema (16,17) in the first admission and presence of severe metabolic acidosis, hypocalcaemia, and severe pneumonia contributed in developing sclerema (1) in the second admission. Thus, our observation of two consecutive episodes of sclerema in successive hospitalizations in this case is understandable.

Sclerema is an ominous prognostic sign. With this rare but dismal skin condition, it is widely believed that death is almost inevitable in majority of cases of sclerema despite intensive management (1,5). Unlike our case infant, the majority of cases die within hours or days of the onset of the condition, regardless of intensive treatment (1,2). Septic shock is the most significant predictor of death probably due to the compromised and ineffective peripheral circulation (4,11). Prematurity, hypothermia, hypoxia, abdominal distension, and pneumonia have been found to be other factors associated with a high fatality rate in scleremic infants (4). Although our case infant had most of these poor prognostic factors, prompt and aggressive circulatory support, including repeated but meticulous blood transfusions, correction of hypoxia and metabolic disturbances, appropriate antibiotics and other intensive care support could have played a role in the positive outcome in our case infant. Transfusion of whole human blood has been reported to improve survival of infants with sclerema (4,11), although the actual mechanism of improvement is not well-understood. In our case, on both the occasions, sclerema started to disappear dramatically after initiation of transfusion of whole human blood but full recovery from sclerema required repeated transfusions. The observation of the beneficial effect of blood transfusion has been reported previously from icddr,b (1,4).

In this case, sclerema was diagnosed clinically. We considered three other skin conditions before making this diagnosis, which were subcutaneous fat necrosis, pseudoscleroderma, and scleroderma of Buschke (1,18). We did not perform a skin biopsy to diagnose sclerema because of the lack of parental consent for the procedure, and x-ray diffraction technique—the gold standard for detecting triglyceride crystals—is not available in Bangladesh.

### Conclusions

Clinicians should be vigilant to look for fatal sclerema in severely-malnourished young infants with overwhelming sepsis even in successive hospitalizations, and such young infants should be managed with blood transfusion, broad-spectrum antibiotics, correction of electrolyte imbalance, in addition to other routine management of malnutrition. The events underscore the importance of urgent need for continuous nutritional follow-up in the community following discharge after recovery from acute illness in order to prevent the relapse of deadly sclerema and simultaneously increase the survival rate in such children.
